# Secure Multi-Directional Independent Transmission Based on Directional Modulated 2D Conformal Phased Array

**DOI:** 10.3390/s25226882

**Published:** 2025-11-11

**Authors:** Fulin Wu, Pengfei Zhang, Yangzhen Qin, Xiaoyang Gong, Hongmin Lu

**Affiliations:** The School of Electronic Engineering, Xidian University, Xi’an 710071, China; fulinwu@stu.xidian.edu.cn (F.W.); zhangpf@mail.xidian.edu.cn (P.Z.); 22021110257@stu.xidian.edu.cn (Y.Q.); 25021211788@stu.xidian.edu.cn (X.G.)

**Keywords:** Multi-directional Independent Transmission (MIT), Directional Antenna Modulation (DAM), conformal phased array

## Abstract

Directional Antenna Modulation (DAM) utilizing 2D conformal phased arrays has been demonstrated to enable secure Multi-directional Independent Transmission (MIT) over a broad angular range. This paper proposes an unbalanced DAM technique that dynamically allocates power according to transmission distance, thereby significantly enhancing transmission efficiency in practical scenarios where receivers are located at varying distances. In particular, a high-efficiency Differential Evolution (DE) optimization algorithm integrated with an “alien species invasion” mechanism is developed to accelerate convergence and optimize the phase delays of each array element. Bit Error Rate (BER) analysis for MIT reveals superior directional security compared to traditional methods, with conformal arrays providing wider angular coverage and spherical sparse arrays overcoming the half-wavelength spacing limitation. The simulation results validate that the proposed system achieves simultaneous secure transmissions in multiple directions while maintaining a BER below −40 dB.

## 1. Introduction

Conformal phased arrays are widely utilized in radio transmission systems due to their flexible integration capabilities and robust beam-steering performance, making them well-suited for deployment on the surfaces of irregularly shaped devices. However, traditional phased array systems face several key limitations. Their transmission speed is constrained by the system bandwidth [1]. More critically, they lack inherent physical-layer security; although the signal magnitude and phase vary spatially, the baseband information remains identical across different directions [2]. Additionally, the energy of an array is typically focused in a single direction, preventing the support of simultaneous Multi-directional Independent Transmission (MIT) [3].

Directional Antenna Modulation (DAM) [4,5,6,7,8,9] has emerged as a promising solution to overcome these limitations. Unlike baseband modulation, where information is modulated before transmission, DAM directly modulates the radio frequency antenna’s radiation field using the transmitted data itself.

DAM technique was first proposed by V. F. Fusco [4], who implemented baseband data encoding by constructing a microstrip patch antenna on a silicon substrate to achieve the amplitude modulation of electromagnetic waves. M.H. Jwair introduced a reconfigurable metasurface layer (RMSL)-loaded microstrip antenna, where the antenna gain was dynamically adjusted by switching between two RMSL states, enabling direct electromagnetic signal modulation [5]. Subsequent studies [6,7,8] demonstrated that DAM can enhance the system bandwidth and increase data transmission rates. Additionally, Reference [9] conducted a comprehensive analysis of radiation efficiency in DAM-based transmitters and derived the theoretical efficiency gain bounds for the technology.

In recent years, the direct modulation antenna has been widely studied as a method to improve physical layer security. Directional Modulation (DM) involves summing the far-field patterns of different array elements in various directions to transmit constellation symbols in a specific direction, while distorting the constellation in other directions [10]. Several DM beamforming approaches have been developed, including switching methods [11,12,13], dual-beam superposition [14,15], reconfigurable attenuators or phase shifters [16,17,18], and vector modulators [19]. Notable implementations include Babakhani’s NFDAM-based 60 GHz directional security transmitter, which uses an active dipole surrounded by switch-controlled parasitic scatterers [11], Daly and Bernhard’s four-element phased array with pattern-reconfigurable capability [16], and a vector-based DM system representation that enables an improved transmitter design [19]. Recent innovations have addressed system complexity by using Discrete Fourier Transform (DFT)-based fixed matrix generation for multi-directional transmission [20] and compact antenna solutions, such as Narbudowicz’s dual-antenna system combining monopole and dielectric patch antennae [21], Parron’s dual-layer stacked patch antenna generating four radiation modes for full azimuthal coverage [22], and Zandamela’s triple-layer circular patch antenna employing spherical mode analysis [23], alongside smartwatch-integrated three-port designs [24]. For enhanced security against eavesdropping through inverse mapping, Lu et al. proposed mmWave power amplifier sub-symbol modulation to create non-repeating, time-varying constellation distortions in unintended directions [25]. This marks a significant advancement in DM, transitioning from a theoretical concept to a practical implementation for secure wireless communications.

However, most reported multi-directional transmission systems rely on 1D linear phased arrays and often assume isotropic radiating elements, which makes them unsuitable for practical Multi-directional Independent Transmission (MIT) applications due to three key limitations: (1) 1D arrays only support beam steering in a single dimension, whereas, in practical scenarios, receivers can be arbitrarily positioned in 3D space; (2) the achievable angular separation between secure beams is severely limited; and (3) the common scenario of receivers located at varying distances is rarely addressed, nor are the directional radiation characteristics of practical antenna elements considered.

This paper addresses these limitations by utilizing 2D conformal arrays to enhance the performance of linear-array-based DAM systems. We propose an unbalanced DAM technique to improve the MIT efficiency for receivers at varying distances, where “unbalanced” refers to the non-uniform power allocation across transmissions to different distances. The Bit Error Rate (BER) calculation method for MIT is systematically discussed, and a high-efficiency Differential Evolution (DE) optimization algorithm is developed to determine the necessary phase delays of each array element for DAM implementation.

The structure and main contributions of this paper are as follows. Section 2 and Section 3 separately explain the principle of DAM and MIT. Section 4 presents the BER calculation method for MIT. Section 5 describes the improved DE optimization algorithm. The improvement of DAM by using a 2D conformal array and unbalanced DAM technique are discussed in Section 6, and the simulation results are shown in Section 7. A spherical conformal array is used in DAM in Section 8. Finally, Section 9 summarizes the key contributions of this work.

## 2. DAM for Phased Array

**Figure 1:**
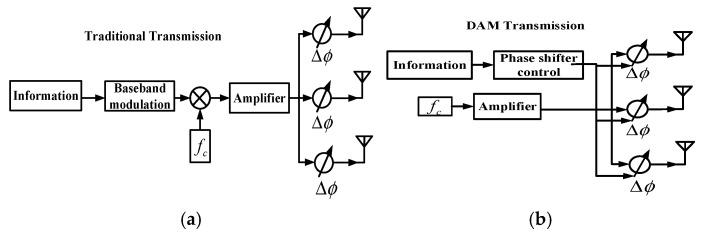
(**a**) Transmission based on traditional method. (**b**) Transmission based on DAM.

The transmission schemes for both traditional methods and DAM are illustrated in Figure 1a,b [16].

Unlike traditional phased arrays, where baseband modulation limits the system’s bandwidth, Directional Antenna Modulation (DAM) directly modulates the radiation pattern using phase shifters controlled by the transmitted data. This approach enables a wider effective bandwidth and provides inherent directional security [16].

The complex amplitude of the electric field at the receiving points can be expressed as follows:

(1) $$E_{r}\left(\theta ,\varphi \right)=\sum_{i=1}^{N}\frac{E_{0}}{4\pi \varepsilon_{0}r_{i}}F_{i}(\theta ,\varphi )(A_{i}e^{j\phi_{i}})e^{-jkr_{i}\left(\theta ,\varphi \right)}$$

where $r_{i}(\theta ,\varphi )$ is the distance from the center of element *i* to the receiving point, and $F_{i}(\theta ,\varphi )$ is a direction function. $\phi_{i}$ is the phase shift of element *i*. $A_{i}$ is the amplitude weighting factor and is always set to 1.

**Figure 2:**
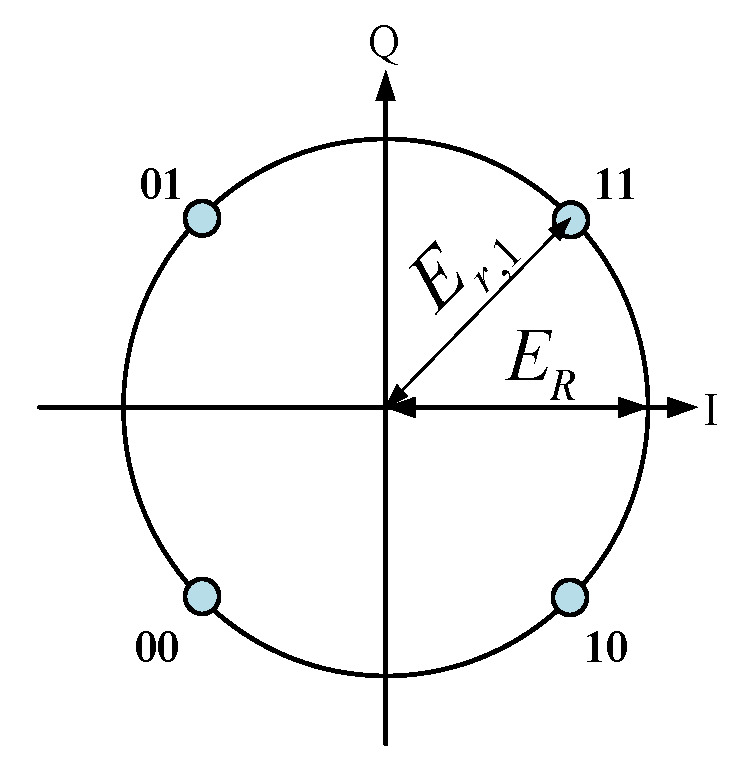
Constellation of QPSK.

Consider the Quadrature Phase Shift Keying (QPSK) modulation method, where two bits of the digital information are grouped together and represented by a signal. The four kinds of two-bit signal, “11,01,00,10”, are presented by the four points $E_{R}e^{j(m*45^{\circ })},m=1,2,3,4$ in the constellation diagram, as shown in Figure 2. In the DAM system, when a two-bit signal is transmitted, we take “11” as an example. The transmitter selects a set of phase shifts, denoted as $\phi_{i}^{1}(i=1∼N)$, for all elements to ensure that the complex amplitude of the receiving field, represented by $E_{r}$, equals a desired value, $E_{R}e^{j45^{\circ }}$. We denote the receiving field as $E_{r}^{1}$, which satisfies the condition $E_{r}^{1}=E_{R}e^{j(45^{\circ })}$. When the receiver observes the fields corresponding to $E_{R}e^{j(45^{\circ })}$, it interprets the received information as ‘11’. The same approach is used to transmit the other three two-bit signals. To achieve the received fields $E_{r}^{m}$ at the destination, which are equal to $E_{R}e^{j(m*45^{\circ })},m=1,2,3,4$, the phase shifts configurations $\phi_{i}^{m},m=1,2,3,4$ are applied to all elements at the transmitter. That is,

(2) $$E_{r}^{m}=E_{R}e^{j(m*45^{{}^{\circ}})},m=1,2,3,4$$

Equation (2) defines the received fields for the four transmission states, enabling the sequential transmission of two-bit symbols by switching the array’s phase configurations. Clearly, the phase shifts of all elements are directly controlled by the transmitted data.

The direction-dependent security of DAM stems from the directional variation of the received field, as described in Equation (1). Even at equal distances from the array center, both the amplitude and phase of the received field vary significantly with direction.

In conventional transmission systems, information recovery relies solely on amplitude. Eavesdroppers can, therefore, compensate for amplitude attenuation using high-sensitivity receivers. However, in DAM, decoding depends on the phase of the received signal. Since phase distortion cannot be easily corrected, eavesdroppers in undesired directions are unable to recover the transmitted information.

**Figure 3:**
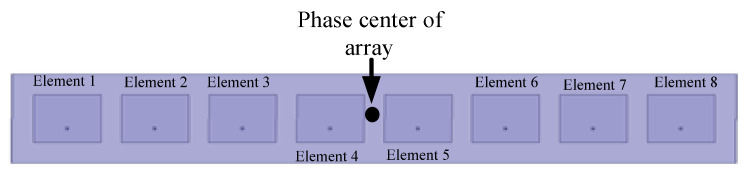
Eight-element array.

**Figure 4:**
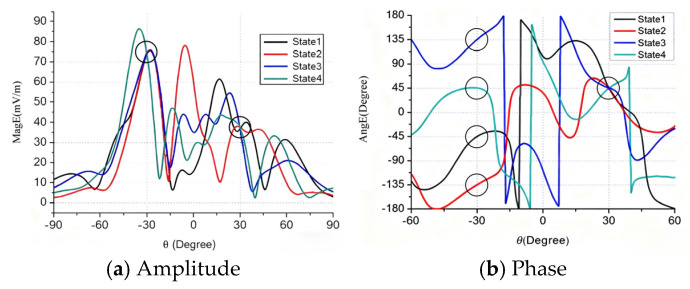
The radiation pattern of eight-element array of four states.

Consider an eight-element patch array shown in Figure 3. The spacing between adjacent antenna elements is λ/2 and the phase center is the center of the array. The direction of $\theta =-30^{\circ }$ is chosen as the intended transmission direction. Table 1 offers the phase shifts of eight elements in four states $\left(A_{i}=1\right)$. Figure 4 shows the radiation pattern of the array in such four states about amplitude and phases. The black circles indicate the radiation pattern of the array in the given direction. For the given direction, the amplitudes of four states are equal, and the phases of them are 45°, 135°, 225°, and 315° separately. The receiver in this direction can recover the information correctly, while, in other directions, the phases of fields are quite scrambled and the recovering of information will be very difficult, or even impossible. An extreme case appears in the direction of $\theta =30^{\circ }$, where the phases of fields for four states are equal to each other, and no information can be recovered from the fields. Hence, the DAM provides a high directional-dependent security.

## 3. MIT Based on DAM

**Figure 5:**
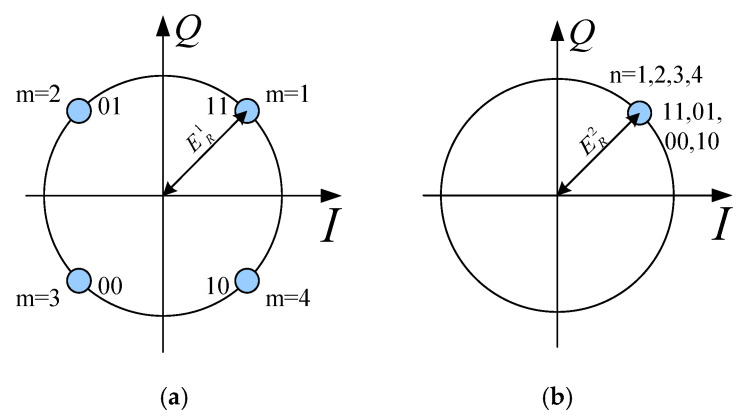
(**a**) Constellation of Direction 1. (**b**) Constellation of Direction 2.

The field constellation for $\theta =-30^{\circ }$ is shown in Figure 5a, where the four states correspond to symmetrical points in the I/Q plane. For $\theta =30^{\circ }$, the fields of the four states are virtually identical, as demonstrated by the complete overlap of their constellation points at a single location, marked by a red marker in Figure 5b. This overlap results from a careful selection of the four sets of phase delays.

The four sets of phase delays in Table 1 form group one, and are denoted by $\phi_{i}^{m,n},i=1∼N,m=1∼4,n=1$ (where *i* represents the number of elements, *m* is the number of points in constellation of $\theta =-30^{\circ }$, and *n* is the number of points in constellation of $\theta =30^{\circ }$). The corresponding radiation fields can be expressed as $E_{r}^{m,n}(\theta =-30^{\circ })$ for m=1∼4,n=1. Similarly, the remaining three groups of phase delays, denoted as $\phi_{i}^{m,n}$, can be derived. The four groups of phase delays consist of 16 sets of $\phi_{i}^{m,n},i=1∼N,m=1∼4,n=1∼4$, which will lead to the results given by Formula (3):

(3) $$\left\{\begin{array}{l} E_{r}^{m,n}{|}_{\theta =-30^{\circ }}=E_{R}^{1}e^{jm*45^{\circ }}\\ E_{r}^{m,n}{|}_{\theta =30^{\circ }}=E_{R}^{2}e^{jn*45^{\circ }} \end{array}\right.$$

Formula (3) serves as the foundation for multi-directional transmission. Consider the case where two series of two-bit signals are transmitted simultaneously in two different directions.

For any fixed m satisfying $E_{r}^{m,n}(\theta =-30^{\circ })=E_{R}^{1}e^{j(m*45^{\circ })}$, there exist four phase-shift configurations $\phi_{i}^{m,n}$ within the total 16 sets that simultaneously satisfy $E_{r}^{m,n}(\theta =30^{\circ })=E_{R}^{2}e^{j(n*45^{\circ })}$ for n=1∼4, and vice versa. This implies that, when transmitting a signal in one direction with the required radiation field $E_{R}^{1}e^{j(m*45^{\circ })}$ (where *m* can be any valve between 1 to 4), an appropriate phase-shift configuration can be selected from four sets to achieve the target field $E_{R}^{2}e^{j(n*45^{\circ })}$ (where *n* can be any value between 1 to 4) in another direction. Thus, the two-directional simultaneous transmissions by one phased array can be realized. This technique can be easily extended to MIT.

## 4. BER of MIT

In practice, two key factors affect BER. One factor is the accuracy of the phase shift, which leads to discrepancies between $E_{r}^{m}$ and $E_{R}e^{j(m*45^{\circ })},m=1,2,3,4$. Another factor is the impact of noise caused by the field distortion.

For the single-direction transmission, the received fields (considering noise) can be rewritten as follows:

(4) $${E_{r}^{\prime }}^{m}=E_{r}^{m}+\left|E_{noise}\right|e^{j\theta_{n}}$$

Here, $E_{noise}=\left|E_{noise}\right|e^{j\theta_{n}}$ represent the effect of noise. Typically, $E_{noise}$ is a random complex value that follows a normal distribution within the region of $\left[0,2\pi \right]$. $\left|E_{noise}\right|$ is distributed within the region of [0,∞], and its probability density function is a Gauss function with a mean value of 0, and a standard deviation of $N_{0}/2$. $N_{0}$ is the power density of the noise.

**Figure 6:**
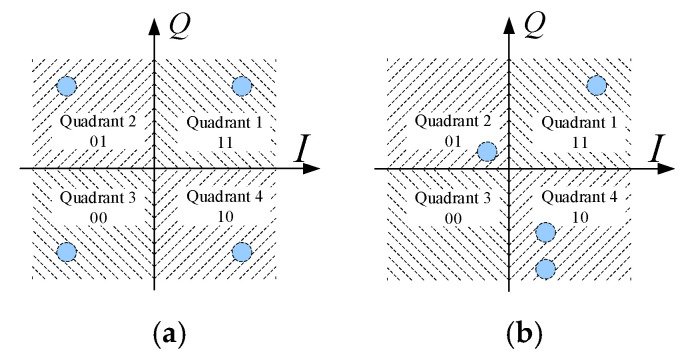
(**a**) Classical constellation. (**b**) Distorted constellation.

As a result, the noise-corrupted received field points deviate significantly from the four ideal points, denoted as $E_{R}e^{j(m*45^{\circ })}$, in the I/Q plane. The Maxim Likelihood Method [26] is commonly used for information recovery: the distances from ${E_{r}^{\prime }}^{m}$ to the four points of $E_{R}e^{j(m*45^{\circ })},m=1,2,3,4$ are calculated, and the closest ideal point is selected as the decoded symbol. An equivalent approach partitions the I/Q plane into four decision regions (Figure 6a), each corresponding to a unique two-bit signal. The received signal is recovered by determining which region ${E_{r}^{\prime }}^{m}$ falls into. In some cases, the distinction between $E_{r}^{m}$ and $E_{R}e^{j(m*45^{\circ })},m=1,2,3,4$ is very large, resulting in a scrambled location for $E_{r,m}$ in the constellation, as shown in Figure 6b.

Both noise interference and phase shift inaccuracies can cause the received field ${E_{r}^{\prime }}^{m}$ to deviate from its intended decision region, leading to demodulation errors. The Bit Error Rate (BER) is defined as the probability of such errors.

For the single-direction transmission, four phase delay sets are used to enable the receiver to decode the four two-bit symbols. The process for estimating the BER involves the following steps:

(1) Calculate all $E_{r}^{m}$ at the receiving points based on the four sets of phase delays.
(2) Rotate $E_{r}^{m}$ as a whole to let the phase of $E_{r}^{1}$ be equal to $45^{\circ }$. (This compensation is necessary because electromagnetic wave propagation induces constellation rotation in practical systems. But, by using the difference QPSK [26], the information is only decided by the relativity location of received fields ${E_{r}^{\prime }}^{m}$ in the constellation. Thus, four states of fields can be rotated to match the reference constellation in Figure 5a.)
(3) Adding the affection of noise by Formula (4).
(4) Calculate the possibility of ${E_{r}^{\prime }}^{m}$ being deviated to the wrong region.
(5) Calculate the BER according to the possibilities’ results.

According to [4], the formula of the error rate of signal for traditional transmission is (5):

(5) $$P_{e}^{s}=\frac{1}{M}\sum_{i=1}^{M}\sum_{k=1,k\neq i}^{M}Q(\frac{d_{ik}/2}{\sqrt{N_{0}/2}})$$

For QPSK, M = 4, $d_{ik}$ is the distance from $E_{r}^{i}$ to $E_{r}^{k}$:

(6) $$Q(x)=\frac{1}{2}erfc(\frac{x}{\sqrt{2}}),erfc(x)=\frac{2}{\sqrt{\pi }}\int_{x}^{\infty }e^{-t^{2}}dt=1-erf(x)$$

As will be discussed later, for the traditional transmission $E_{r}^{m}\approx E_{R}e^{j(m*45^{\circ })}$, the approximation of Formula (5) is as follows:

(7) $$P_{e}^{s}\approx Q(E_{R}/\sqrt{N_{0}})$$

Formulae (5) and (7) are the error rate of the signal. By using Gray coding, the Bit Error Rate (BER) is nearly half of the error rate of the signal [23] and is equal to the following:

(8) $$P_{e}=\frac{1}{2}Q(E_{R}/N_{0})$$

However, Formulae (5)–(8) are not applicable to DAM-based transmission due to the direction-dependent nature of the DAM constellation.

In traditional transmission, I/Q modulation is performed prior to the phase delaying. Therefore, even if the amplitude of $E_{r}^{m}$ becomes very small in other directions, the order and the angular separation between the four points of $E_{r}^{m}$ in the constellation remain unchanged with direction. Formulae (5)–(7) are only applicable in such a scenario. In contrast, for DAM, the order and the angular separation between the four points of $E_{r}^{m}$ in the constellation change significantly with direction, as previously mentioned.

Nonetheless, the five-step BER estimation process remains valid for DAM, and the signal error rate can be derived by analyzing the probability of ${E_{r}^{\prime }}^{m}$ deviating into the incorrect decision regions [26]. Two cases are considered:

(1) ${E_{r}^{\prime }}^{m}$ located in Quadrant m.

Taking the symbol vector that represents “11” in Quadrant 1 as an example, the magnitude and phase of the receiving field could be denoted by $\left|E_{r}^{1}\right|$ and $\theta_{1}$. The distance from the end point of Vector $E_{r}^{1}$ to the boundary between the left and right half planes is $ER_{r}^{1}=\left|E_{r}^{1}\right|\cos(\theta_{1})$. If the power spectral density of the noise is *N*_0_/2, the possibility of ${E_{r}^{\prime }}^{1}$ entering into Quadrant 2 or Quadrant 3 is as follows:

(9) $$P_{1}^{R}=Q\left(\left|ER_{r}^{1}\right|/\sqrt{N_{0}/2}\right)$$

Similarly, the possibility of ${E_{r}^{\prime }}^{1}$ entering into Quadrant 3 or Quadrant 4 is as follows:

(10) $$P_{1}^{I}=Q\left(\left|EI_{r}^{1}\right|/\sqrt{N_{0}/2}\right)$$

where $EI_{r}^{1}=\left|E_{r}^{1}\right|\sin(\theta_{1})$. Thus, the error rate of the signal that represents the possibilities of ${E_{r}^{\prime }}^{1}$ falling into Quadrant 2, Quadrant 3, and Quadrant 4 should be $\frac{Quadrant2}{P_{1}^{R}(1-P_{1}^{I})}$, $\frac{Quadrant3}{P_{1}^{R}P_{1}^{I}}$, and $\frac{Quadrant4}{(1-P_{1}^{R})P_{1}^{I}}$, respectively.

Note that the BER is not the summation of the error rate of the signal. The reason is that, if the symbol vector ${E_{r}^{\prime }}^{1}$ falls into Quadrant 2, the sent message of ‘11’ will be decoded as ‘01’, with one of two bits being not correct; thus, the contribution of BER is only half of $\frac{Quadrant2}{P_{1}^{R}(1-P_{1}^{I})}$. This is also true when ${E_{r}^{\prime }}^{1}$ falls in Quadrant 4. However, if ${E_{r}^{\prime }}^{1}$ falls in Quadrant 3, the recovered information is ‘00’, and the contribution of BER is $\frac{Quadrant3}{P_{1}^{R}P_{1}^{I}}$. Therefore, when ‘11’ is chosen to be sent, the BER is as follows:

(11) $$Error_{11}=\frac{1}{2}\frac{Quadrant2}{P_{1}^{R}(1-P_{1}^{I})}+\frac{Quadrant3}{P_{1}^{R}P_{1}^{I}}+\frac{1}{2}\frac{Quadrant4}{(1-P_{1}^{R})P_{1}^{I}}=\frac{1}{2}\left(P_{1}^{R}+P_{1}^{I}\right)$$

Considering that the possibility of ‘11’ being sent during communication is 1/4, the BER should be the averaged value of four BERs for the cases of ‘11’, ‘01’, ‘10’, and ‘00’ sent.

(12) $$P_{e}=\frac{1}{4}(Error_{11}+Error_{10}+Error_{01}+Error_{00})=\frac{1}{4}\sum_{m=1}^{4}\frac{1}{2}(P_{m}^{R}+P_{m}^{I})$$

(13) $$P_{m}^{R}=Q(\left|ER_{r}^{m}\right|/\sqrt{N_{0}/2}),P_{m}^{I}=Q(\left|EI_{r}^{m}\right|/\sqrt{N_{0}/2})$$

(2) ${E_{r}^{\prime }}^{m}$ not located in Quadrant *m*.

Taking the symbol vector that represents “11” in Quadrant 1 as example, if $ER_{r}^{1}<0$, the possibility of ${E_{r}^{\prime }}^{1}$ being located in Quadrant 2 or Quadrant 3 is that the symbol vector does not pass the boundary between the left and right half planes. It should be $P_{1}^{R}=1-Q\left(\left|ER_{r}^{1}\right|/\sqrt{N_{0}/2}\right)$. Hence, all one needs to do is replace $P_{1}^{R}=Q\left(\left|ER_{r}^{1}\right|/\sqrt{N_{0}/2}\right)$ in Case 1 with $P_{1}^{R}=1-Q\left(\left|ER_{r}^{1}\right|/\sqrt{N_{0}/2}\right)$. It is the same with $P_{1}^{I}$.

It is noteworthy that the relationship in which the BER of the Gray code is approximately half that of the signal is also invalid for DAM. Taking all factors into consideration, the BER for DAM is as follows:

(14) $$p_{e}=\frac{1}{4}\sum_{m=1}^{4}\frac{1}{2}(P_{m}^{R}+P_{m}^{I})$$

(15) $$P_{m}^{R}=\left\{\begin{array}{c} \begin{array}{l} Q(\frac{\left|ER_{r}^{m}\right|}{\sqrt{N_{0}/2}}),[(ER_{r}^{m}>0),(m=1,4)]\\ or[(ER_{r}^{m}<0),(m=2,3)] \end{array}\\ \begin{array}{l} 1-Q(\frac{\left|ER_{r}^{m}\right|}{\sqrt{N_{0}/2}}),[(ER_{r}^{m}<0),(m=1,4)]\\ or[(ER_{r}^{m}>0),(m=2,3)] \end{array} \end{array}\right.$$

(16) $$P_{m}^{I}=\left\{\begin{array}{c} \begin{array}{l} Q(\frac{\left|EI_{r}^{m}\right|}{\sqrt{N_{0}/2}}),[(EI_{r}^{m}>0),(m=1,2)]\\ or[(EI_{r}^{m}<0),(m=3,4)] \end{array}\\ \begin{array}{l} 1-Q(\frac{\left|EI_{r}^{m}\right|}{\sqrt{N_{0}/2}}),[(EI_{r}^{m}<0),(m=1,2)]\\ or[(EI_{r}^{m}>0),(m=3,4)] \end{array} \end{array}\right.$$

$ER_{r}^{m},EI_{r}^{m}$ are the real and imagined parts of $E_{r}^{m}$. The $E_{r}^{m}$ value was obtained from Step 2 as described above. It can be seen that Formulae (5)–(7) are the approximation of a special case of Formulae (14)–(16).

Now, let us focus on another question: how to calculate the BER for MIT. Consider two-direction communication as an example: 16 sets of phase shifts, denoted as $\phi_{i}^{m,n}$(*i* = 1~N, with N being the number of elements, *m* = 1~4, and *n* = 1~4), should be prepared. The fields of the array using phase shifts of $\phi_{i}^{m,n}$ should satisfy Formula (17) in the two directions of $\left(\theta_{1},\varphi_{1}\right),\left(\theta_{2},\varphi_{2}\right)$.

(17) $$\left\{\begin{array}{l} E_{r}^{m,n}\left(\theta_{1},\varphi_{1}\right)=\sum_{i=1}^{N}\frac{E_{0}}{4\pi \varepsilon_{0}r_{i}}F_{i}(A_{i}e^{j\phi_{i}^{m,n}})e^{-jkr_{i}}\approx E_{R}^{1}e^{j(m*90^{\circ }+\Phi_{1})}\\ E_{r}^{m,n}\left(\theta_{2},\varphi_{2}\right)=\sum_{i=1}^{N}\frac{E_{0}}{4\pi \varepsilon_{0}r_{i}}F_{i}(A_{i}e^{j\phi_{i}^{m,n}})e^{-jkr_{i}}\approx E_{R}^{2}e^{j(n*90^{\circ }+\Phi_{2})} \end{array}\right.$$

$\Phi_{1}$ and $\Phi_{2}$ could be any values.

For DM-based MIT, two key aspects must be emphasized. First, for each fixed m in (17), there are four sets of phase shifts for *n* = 1~4. Thus, the BER should be calculated as the average of the four $P_{e}$. These four $P_{e}$ are obtained by substituting $E_{r}^{m}$ in Formulae (14)–(16) with $E_{r}^{m,n}$ (*n* = 1~4), respectively. Second, two series of information are transmitted simultaneously in two different directions. By treating these two series of information as the receiving data, two BERs can be calculated. Hence, the BERs for the two-directional transmission is follows:

(18) $$P_{e}^{i}=\frac{1}{4}\sum_{m=1}^{4}\frac{1}{2}\left(\sum_{n=1}^{4}P_{i,m,n}^{R}+P_{i,m,n}^{I}\right)$$

(19) $$P_{i,m,n}^{R}=\left\{\begin{array}{c} \begin{array}{l} Q(\frac{\left|ER_{r}^{m,n}\right|}{\sqrt{N_{0}/2}}),[(ER_{r}^{m,n}>0),(T=1,4)]\\ or[(ER_{r}^{m.n}<0),(T=2,3)] \end{array}\\ \begin{array}{l} 1-Q(\frac{\left|ER_{r}^{m,n}\right|}{\sqrt{N_{0}/2}}),[(ER_{r}^{m,n}<0),(T=1,4)]\\ or[(ER_{r}^{m,n}>0),(T=2,3)] \end{array} \end{array}\right.$$

(20) $$P_{i,m,n}^{I}=\left\{\begin{array}{c} \begin{array}{l} Q(\frac{\left|EI_{r}^{m,n}\right|}{\sqrt{N_{0}/2}}),[(EI_{r}^{m,n}>0),(T=1,2)]\\ or[(EI_{r}^{m.n}<0),(T=3,4)] \end{array}\\ \begin{array}{l} 1-Q(\frac{\left|EI_{r}^{m,n}\right|}{\sqrt{N_{0}/2}}),[(EI_{r}^{m,n}<0),(T=1,2)]\\ or[(EI_{r}^{m,n}>0),(T=3,4)] \end{array} \end{array}\right.$$

For the information transmitted to Direction 1, *i* = 1 and *T* = *m*. For the information transmitted to Direction 2, *i* = 2 and *T* = *n*. $ER_{r}^{m,n}$, $EI_{r}^{m,n}$ are the real and imaginary parts of $E_{r}^{m,n}$. The calculation method of BER can be extended to the cases of three- or multi-directional communication.

## 5. Optimization Algorithm Based on DE

For the one-direction transmission, the objective is to determine the appropriate $\phi_{i}^{m},m=1\sim 4$ that ensures

(21) $$E_{r}^{m}\approx E_{R}e^{j(m*45^{\circ })},m=1,2,3,4$$

For the two-direction transmission, the objective is to determine the appropriate $\phi_{i}^{m,n},i=1∼N,m=1∼4,n=1∼4$ that ensure

(22) $$\left\{\begin{array}{l} E_{r}^{m,n}{|}_{\theta_{1}}\approx E_{R}^{1}e^{j(m*45^{\circ })}\\ E_{r}^{m,n}{|}_{\theta_{2}}\approx E_{R}^{2}e^{j(n*45^{\circ })} \end{array}\right.$$

From Formulae (14)–(16), it can be concluded that a larger value of $E_{R}$ results in a lower ER. Hence, the optimization task involves finding a set of phase delays that maximizes the radius of constellation $E_{R}$ in (21) or (22).

This study employs the Differential Evolution (DE) optimization algorithm due to its advantages over other alternatives such as Genetic Algorithms (GAs) or Particle Swarm Optimization (PSO) [27]. The details of the DE algorithm are omitted for brevity, but the two proposed measures to improve the optimization efficiency are discussed below.

The first measure involves the careful selection of the initial values. Consider the two-direction transmission as an example: 16 sets of phase delays for N elements need to be optimized simultaneously. For optimization in such a large space, a random initial value can lead to unstable convergence and low efficiency.

**Figure 7:**
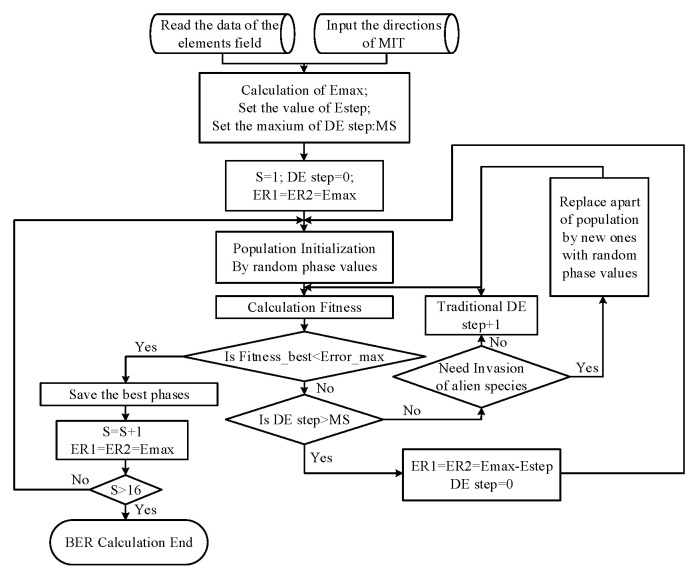
Flowchart of DE.

Inspired by the ecological niche concept, we employ a series of local optimizations to determine suitable initial values. The procedure begins by setting a large fixed initial value for $E_{R}$ in Equation (21). Sixteen phase delay sets are then sequentially optimized, considering only the phase requirements specified in Equation (21). Phase-delay configurations that satisfy these requirements are retained, while non-compliant sets undergo repeated optimization with progressively reduced $E_{R}$ values until all sixteen sets meet the specified phase conditions. These qualified phase-delay sets serve as the initial values for a subsequent global optimization process that identifies the optimal phase-delay combination yielding the best $E_{R}$ performance, as demonstrated in [28]. Figure 7 presents the complete optimization flowchart.

The fitness during DE is defined as (23):

(23) $$Fitness=\max(\left|E_{r}^{m,n}(\theta_{1},\varphi_{1})-E_{R}^{1}e^{j(m*90^{\circ }+\Phi_{1})}\right|,\left|E_{r}^{m,n}(\theta_{2},\varphi_{2})-E_{R}^{2}e^{j(n*90^{\circ }+\Phi_{2})}\right|)-Error$$

As shown in Figure 7, 16 sets of phase shifts are optimized one by one. The phase shifts that satisfy the requirements of Equation (17) are saved. The other sets of phase shifts are optimized by taking a gradually decreasing value of $E_{R}$ until 16 sets of phases are obtained. S in Figure 7 is equal to (m−1)∗4+n.

Another measure to address the slowdown in optimization speed is the method we refer to as the “invasion of alien species”.

As commonly observed in intelligent optimization algorithms, the convergence rate of fitness values typically decreases as the number of iterations increases. Inspired by ecological phenomena, where invasive species drive rapid population adaptation, we propose an enhanced Differential Evolution (DE) algorithm that incorporates an “alien species invasion” mechanism. In this approach, at predetermined intervals during the iterations, a set of randomly generated individuals replaces an equal number of low-fitness population members.

**Figure 8:**
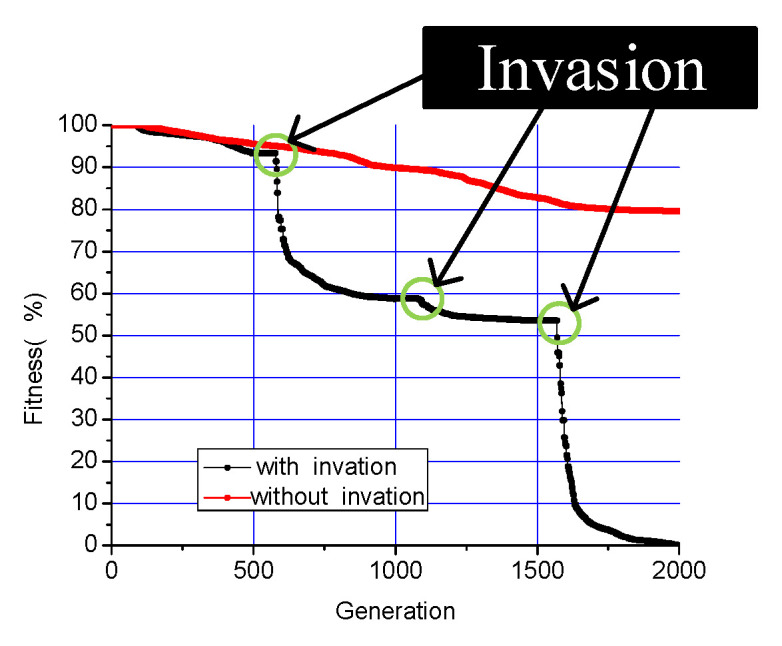
Convergence of DE.

Figure 8 compares the fitness convergence between conventional DE and our modified approach. In the conventional DE, the new individuals replace the original ones every 500 generations, as indicated by the three circles in the figure. The results clearly show that this mechanism effectively prevents premature convergence and maintains a higher rate of fitness improvement throughout the optimization process.

## 6. Improvement of DAM Based on Conformal Array

Although the feasibility of DAM-based MIT has been demonstrated using linear arrays [13], its practical application remains challenging due to several limitations, as previously discussed.

The methods to solve those problems are proposed in this paper.

(1) Since linear arrays can only control the scanning of beams in one dimension, 2D arrays will be employed to provide the capability to steer the beams in two dimensions. Furthermore, a conformal array architecture implemented on curved surfaces is adopted to significantly expand the angular scanning range beyond conventional planar array limitations.
(2) In practical scenarios where receivers are distributed at varying distances, maintaining an equal constellation radius for all directions would require increasing the array’s power output to ensure sufficient energy delivery to the most distant receiver. Therefore, an unbalanced method is proposed to eliminate the need for uniform power boosting by adaptively optimizing constellation radius according to direction-specific transmission distances.

Two methods are very easy to understand; the theme of the unbalanced transmission is illustrated as follows.

For a fixed power of noise, the BER is decided by the radius of constellation $E_{R}$. Thus, the smallest value of $E_{R}^{\min}$ can also be estimated from the highest limitation of BER of $P_{e}^{worst}$.

It is well-known that the amplitude of received fields $E_{R}$ is attenuated with the increase in transmission distance. Consider two receivers in the distances of $r_{1}$ and $r_{2}$: if we hope that the received fields in the two destinations are the same, the relationship of (17) should be satisfied to ensure the BERs of the two directions remain the same (the $N_{0}$ of the two destinations are supposed to be equal):

(24) $$\frac{E_{R,send}^{1}}{r_{1}}=\frac{E_{R,send}^{2}}{r_{2}}=E_{R}^{\min}$$

where $E_{R,send}^{1}$ and $E_{R,send}^{2}$ are the radii of constellation for direction one and two. Rewriting (24) as (25), we obtain the rule of how to set the rate of the radius of constellation for the two directions:

(25) $$\frac{E_{R,send}^{1}}{E_{R,send}^{2}}=\frac{r_{1}}{r_{2}}$$

This rule can also be extended to the MIT for three or more directions.

## 7. Simulation Results

**Figure 9:**
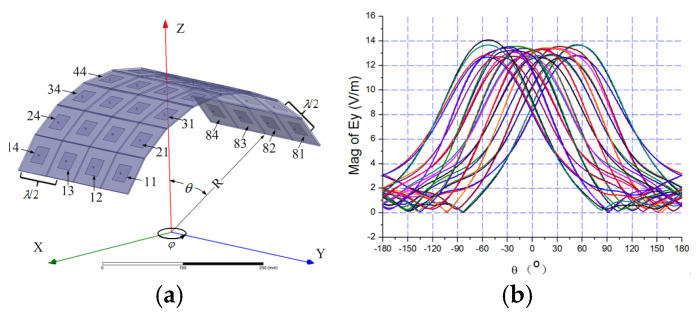
(**a**) Cylinder conformal array. (**b**) Radiation pattern of elements.

To evaluate the performance of the proposed DAM-based MIT system, a 2D cylindrical conformal array (Figure 9) is used for the simulation.

- The radiation character of the conformal array

The 8 × 4 patches are arranged in a cylinder surface as shown in Figure 9a. Four elements are arranged in the y direction and eight elements are arranged in the x direction. The size of each patch is 22.45 mm × 22.45 mm, and the substrate has a dielectric constant of 4.4 with a thickness of 2 mm. All patches work in line polarization (in the direction of y) at 3 GHz. The spacing between antenna elements is λ/2. The radius of cylinder R is 190 mm.

The full-wave electromagnetic simulation software Ansys Electronics Desktop2022 is employed to analyze the radiation character of the array. Figure 9b is the far-field radiation pattern of all elements in the xoz plane.

**Figure 10:**
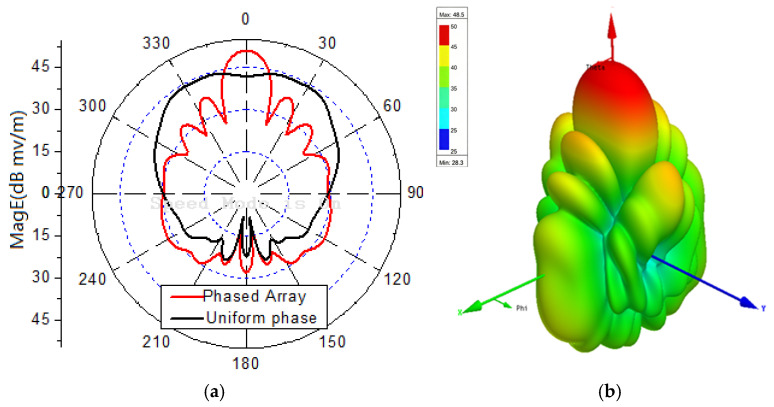
(**a**) Radiation pattern with different phase. (**b**) 3D radiation pattern.

Figure 10a compares the radiation patterns with zero-phase excitation (black line) and phase-shifted excitation (red line) to steer the main lobe to θ=0,φ=0. The red line confirms that the main lobe is successfully steered to the desired direction. Figure 10b shows the 3D radiation pattern for the phase-shifted excitation.

B. MIT of two directions and the security character

Two arbitrary directions $\theta_{1}=25^{\circ }\varphi_{1}=25^{\circ }$ and $\theta_{2}=35^{\circ }\varphi_{2}=135^{\circ }$ are selected for the two-directional transmission. The 16 phase-delay sets are optimized using the enhanced DE algorithm.

**Figure 11:**
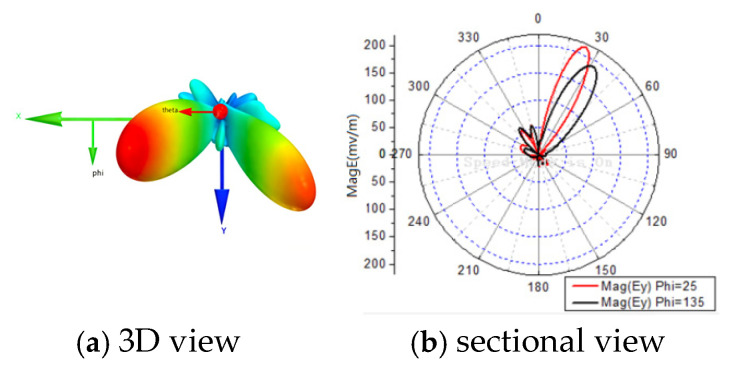
Radiation patterns for two-direction transmission of $\theta_{1}=25^{\circ }\varphi_{1}=25^{\circ },\theta_{2}=35^{\circ }\varphi_{2}=135^{\circ }$.

Figure 11a shows the 3D radiation pattern of the array for one of the 16 phase-delay sets, confirming that energy is focused on the two target directions. Especially, the maximum field amplitude does not necessarily coincide with the target direction, as the optimization objective is to minimize the BER.

**Figure 12:**
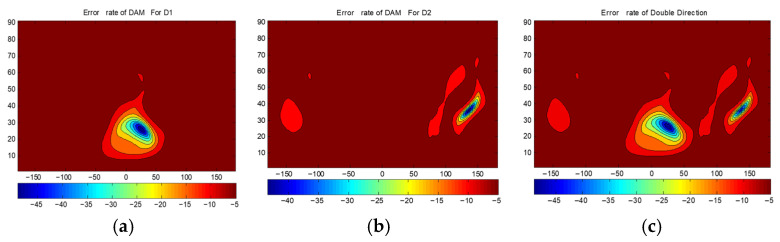
The BERs(dB) of DAM: (**a**) for the information of Direction 1; (**b**) for the information of Direction 2; and (**c**) the combined results.

**Figure 13:**
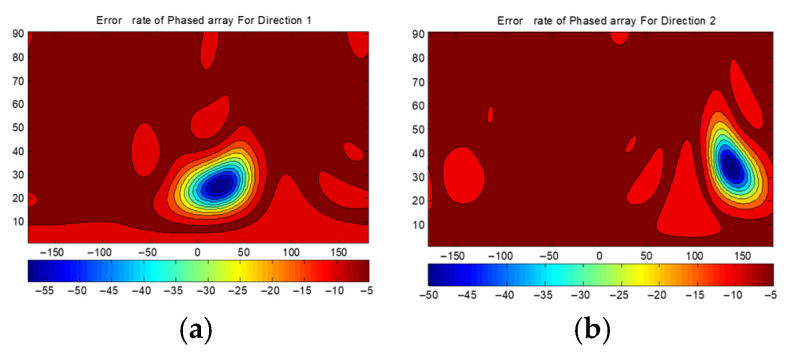
The BERs(dB) of traditional transmission: (**a**) beam steered to Direction 1; and (**b**) beam steered to Direction 2.

**Figure 14:**
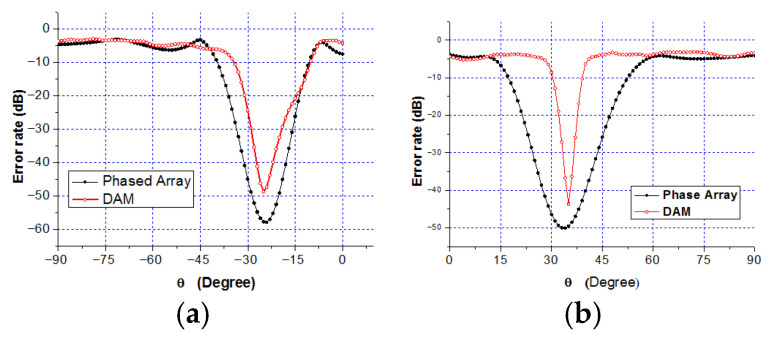
Sectional view BER: (**a**) $\varphi_{1}=25^{\circ }$; and (**b**) $\varphi_{2}=135^{\circ }$.

Figure 12 presents the BER distributions for the two-directional DAM system. Figure 12a is Direction 1 with $\theta_{1}=25^{\circ },\varphi_{1}=25^{\circ }$. Figure 12b is Direction 2 with $\theta_{2}=35^{\circ },\varphi_{2}=135^{\circ }$. Figure 12c shows the combined BER. Figure 13 shows the BER of a traditional phased array steering its beam to Direction 1 and Direction 2. Figure 14 presents the BER sectional view for both system in the xoz planes of $\varphi_{1}=25^{\circ }$ and $\varphi_{2}=135^{\circ }$.

Three conclusions are drawn from Figure 12, Figure 13 and Figure 14:

(1) The BER at the two target directions is extremely low (below −40 dB), confirming that the DAM system enables the simultaneous independent transmission in multiple directions.
(2) The area with the BER less than −40 dB is significantly smaller for the DAM system compared to the traditional phased array, indicating narrower secure beams and superior direction-dependent security.
(3) The BER at the target directions for the DAM system is slightly higher than that of the traditional phased array (which focuses all of the energy on a single direction), but it still meets practical requirements (such as BER < −40 dB). This trade-off is necessary to support simultaneous multi-directional transmission.

C. The unbalance transmission

In the above analyses, the distances for the two directions are supposed to be equal. Now, consider the new case for $r_{1}/r_{2}=0.5$: the 16 sets of phase delay are optimized according to Formula (13).

**Figure 15:**
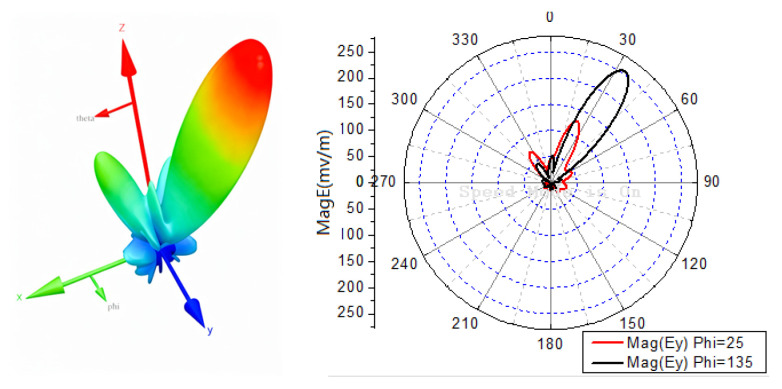
Radiation patterns for two directions’ unbalanced transmissions.

**Figure 16:**
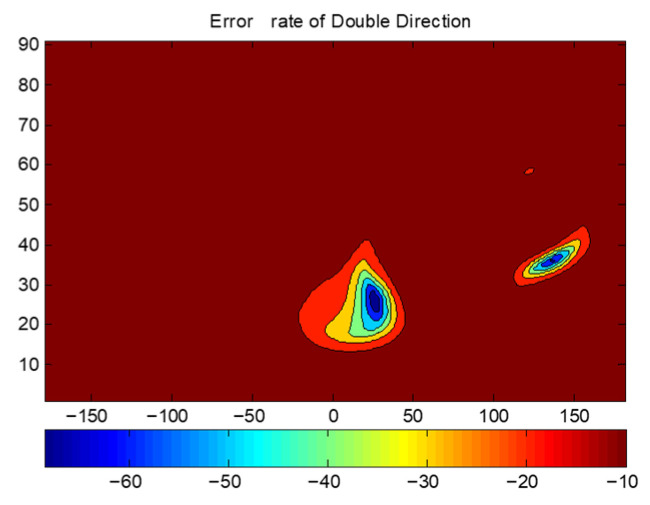
The combined results of BERs(dB) of unbalanced DAM.

Figure 15 shows the radiation pattern for the unbalanced DAM system, confirming that the field amplitude in Direction 1 (the closer receiver) is half that in Direction 2 (the farther receiver)—as expected. Figure 16 presents the combined BER distribution for the unbalanced system, showing that the BER at both directions is maintained at the same level (below −40 dB). Comparing Figure 16 with Figure 12c, the BER of the unbalanced system is reduced by approximately 10 dB for both directions—demonstrating improved energy efficiency. If the same BER requirement is maintained, the unbalanced system can operate at a lower total power compared to the balanced system.

D. Advantage of conformal array

To evaluate the angular coverage advantage of the conformal array, four cylindrical conformal arrays with different radii are tested: (1) R=190 mm, (2) R=160 mm, (3) R=130 mm, and (4) R=∞ (planar array). Two directions $\varphi_{1}=20^{\circ },\varphi_{2}=170^{\circ }$ are selected, with equal transmission distances. The BER is recorded as θ, and varies from $15^{\circ }$ to $70^{\circ }$.

**Figure 17:**
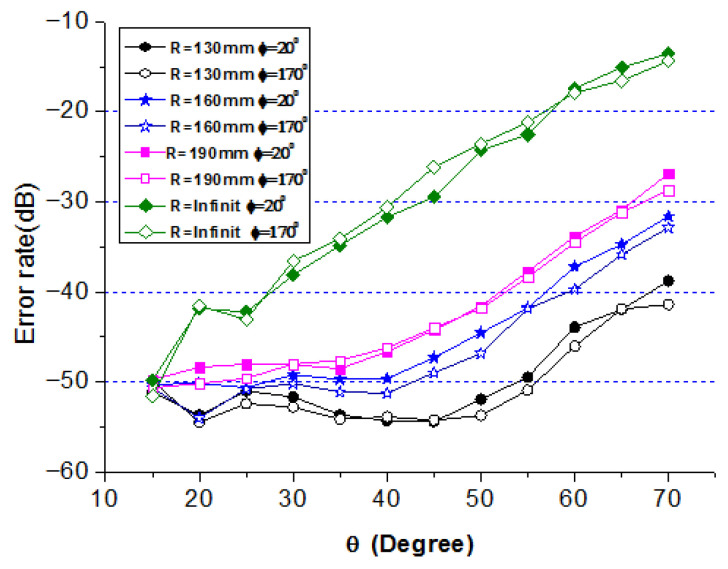
Error rate of cylindrical conformal array for different R.

Figure 17 shows the BER curves for the four arrays. For a strict BER requirement of −50 dB, the planar array (R=∞) can only support $\theta \leq 15^{\circ }$, whereas the conformal array with R=130 mm supports $\theta \leq 55^{\circ }$. This demonstrates that conformal arrays provide a significantly wider angular coverage than planar arrays, with the coverage increasing as the curvature radius decreases. A full-cylinder conformal array could potentially achieve omnidirectional coverage.

## 8. Sparse Spherical Array

Traditional phased arrays require the space between elements to be less than half of the wavelength to avoid the grating lobes. However, this limitation can be overcome in DAM systems for two reasons. The first is that the grating lobes can be avoided during the optimization of phase delays. The second is, even if there are some lobes with a comparably large field amplitude in some directions, the distortion of the phases can cause the BER to be very high in those directions.

**Figure 18:**
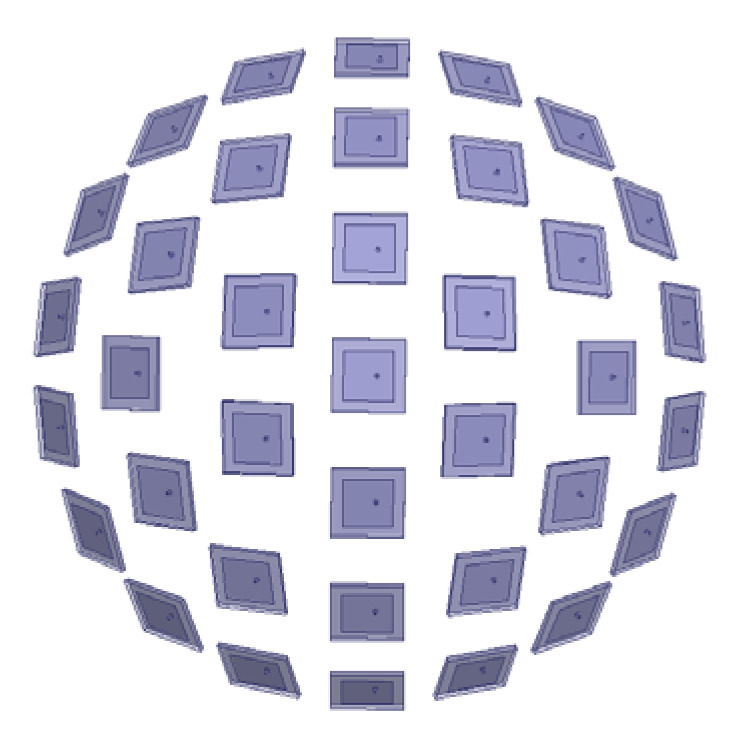
Spherical sparse array.

**Figure 19:**
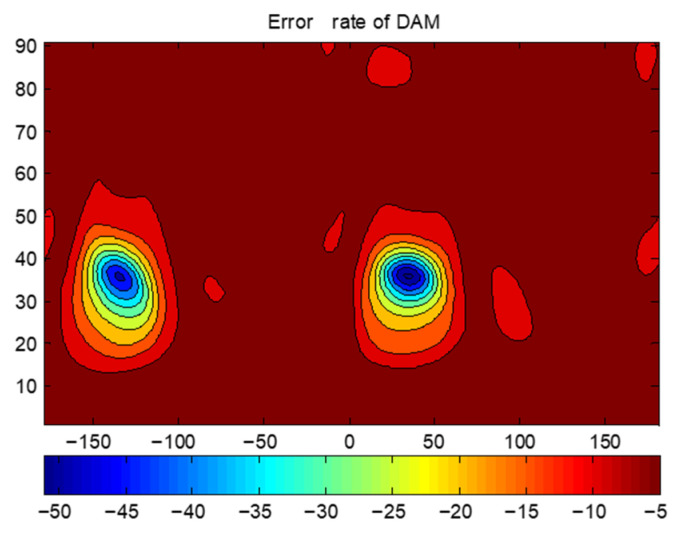
BER of sparse array.

To validate this, a spherical conformal array with 37 elements shown in Figure 18 is tested. The radius of the sphere is 156.7 mm. The distance between the cells is 57.31 mm, greater than the half-wavelength of 3 GHz. The two transmission directions are taken as $\varphi_{1}=35^{\circ },\varphi_{2}=-135^{\circ },\theta_{1}=\theta_{2}=35^{\circ }$. Figure 19 presents the BER distribution of the sparse array, showing no significant grating lobes—confirming that DAM systems can overcome the half-wavelength spacing limitation. This enables the design of compact, low-cost sparse arrays for MIT applications.

## 9. Conclusions

This paper presents a comprehensive analysis of Multi-directional Independent Transmission (MIT) enabled by Directional Antenna Modulation (DAM) in 2D conformal phased arrays. A systematic BER calculation method for single and multi-directional transmission is proposed, and a high-efficiency Differential Evolution (DE) optimization algorithm—integrated with an “alien species invasion” mechanism—is developed to optimize the phase delays of array elements.

The limitations of linear-array-based DAM systems are addressed by utilizing 2D conformal arrays for wide-angle coverage and an unbalanced transmission technique for distance-adaptive power allocation. The feasibility of using sparse arrays in DAM is validated using a spherical conformal array, which overcomes the half-wavelength spacing limitation of traditional phased arrays. The simulation results demonstrate the superior performance of the proposed system: the area where the Bit Error Rate (BER) remains below −40 dB is drastically reduced compared to traditional phased arrays, enhancing direction-dependent security. In addition, under a stringent BER requirement of −50 dB, the cylindrical conformal array achieves a wider angular coverage than a comparable planar array.

The proposed technique holds significant potential for applications in secure wireless multi-directional transmission and high-speed secure communication systems.

## Figures and Tables

**Table 1:** Eight element phase shifts in four states.

Elements	State1	State2	State3	State4
1	180	180	180	−118
2	−97	129	−117	−75
3	−109	6	−9	23
4	78	71	113	86
5	172	180	−99	5
6	−136	−84	174	−92
7	279	62	98	−50
8	−173	180	109	135

## Data Availability

The complete dataset is subject to approval, but relative formulae, phase shifts, and antenna models have been provided in detail in the manuscript and sufficient to support the reproduction of this paper.
